# Post-activation Performance Enhancement after a Bout of Accentuated Eccentric Loading in Collegiate Male Volleyball Players

**DOI:** 10.3390/ijerph182413110

**Published:** 2021-12-12

**Authors:** Kuo-Wei Tseng, Jyun-Ru Chen, Jun-Jie Chow, Wei-Chin Tseng, Giancarlo Condello, Hsia-Ling Tai, Szu-Kai Fu

**Affiliations:** 1 Department of Exercise and Health Sciences, College of Kinesiology, University of Taipei, Taipei 111036, Taiwan; fossil0405@yahoo.com.tw (K.-W.T.); tpeckenny@gmail.com (J.-R.C.); junjiechow928@gmail.com (J.-J.C.); 2 Department of Physical Education, College of Science, University of Taipei, Taipei 100234, Taiwan; speedceng@gmail.com (W.-C.T.); danatai1008@gmail.com (H.-L.T.); 3 Department of Medicine and Surgery, University of Parma, 43126 Parma, Italy; giancarlo.condello@unipr.it; 4 Graduate Institute of Sports Training, College of Kinesiology, University of Taipei, Taipei 111036, Taiwan

**Keywords:** resistance training, eccentric overload, jumping ability, muscle power, potentiating effects

## Abstract

The purpose of this study was to investigate the benefit of post-activation performance enhancement (PAPE) after accentuated eccentric loading (AEL) compared to traditional resistance loading (TR). Sixteen male volleyball athletes were divided in AEL and TR group. AEL group performed 3 sets of 4 repetitions (eccentric: 105% of concentric 1RM, concentric: 80% of concentric 1RM) of half squat, and TR group performed 3 sets of 5 repetitions (eccentric & concentric: 85% of 1RM). Countermovement jump (CMJ), spike jump (SPJ), isometric mid-thigh pull (IMTP), and muscle soreness test were administered before (Pre) exercise, and 10 min (10-min), 24 h (24-h), and 48 h (48-h) after exercise. A two-way repeated measures analysis of variance was used to analyze the data. Peak force and rate of development (RFD) of IMTP in AEL group were significantly greater (*p* < 0.05) than TR group. The height, peak velocity, and RFD of CMJ, height of SPJ, and muscle soreness showed no interaction effects (*p* > 0.05) groups x time. AEL seemed capable to maintain force production in IMTP, but not in CMJ and SPJ. It is recommended the use of accentuated eccentric loading protocols to overcome the fatigue.

## 1. Introduction

Vertical jumping is a critical ability in volleyball and is related to serving, spiking, or blocking. It is widely recognized that muscular strength underpins several motor performances, included jumping [1], hence the practice of resistance training is fundamental for muscular strength development [2]. Traditional resistance training (TR) consists of lifting (concentric) and lowering (eccentric) an identical externally imposed load, and it prescribes equivalent absolute loads for the concentric and eccentric action of an exercise [3,4]. However, this traditional approach might not provide an optimal stimulus during the eccentric phase of lifting [4]. During eccentric muscle contractions, peak force production could be greater than 50% compared with concentric contraction [5]. Therefore, loads encountered in TR exercise are limited by concentric strength, leading trainers or practitioners to seek alternative training methods to increase the strength and force production ability of eccentric muscle action, but also to improve specificity and utilization of stretch-shortening cycle [4]. A strategy to overcome this issue is the accentuated eccentric loading (AEL). AEL involves the prescription of eccentric loads greater than those prescribed for the concentric part of the movements, hence traditional concentric-eccentric resistance loading is performed, but an additional external load is imposed during the eccentric phase [4,6,7]. AEL demonstrated significant increases in the cross-sectional area (CSA) of IIx fibers and shifts to faster myosin heavy chain (MHC) isoforms [8]. These changes were also accompanied by improvements in force and power production [3,8,9].

A phenomenon known as Post-activation potentiation (PAP) [10] indicates the increase of muscle contractile responses due to a prior muscle activity (which is called conditioning activity), with a subsequent enhancement of voluntary performance, which is known as Post-activation performance enhancement (PAPE) [11]. Therefore, pre-training and pre-competition warm-up exercises are designed for athletes to elicit optimal performance [12,13,14,15]. Several studies had suggested that PAP in the warm-up resulted in improving performance during explosive performance, such as jumping [16,17]. The effect of PAP would dissipate over 4–6 min after the PAP-inducing contraction [18]. However, it has been shown that the PAP effects could occur at 3, 6, 9, and 12 min after a conditioning activity [19].

The use of AEL could be considered a conditioning activity to induce PAPE and, therefore, to enhance a subsequent performance. A few studies have demonstrated the enhancement of performance induced by AEL training (i.e., 1 set of 5 repetitions), but without rest between each repetition [20,21,22]. Conversely, some studies showed that AEL training did not acutely enhance a subsequent performance [6,9,23]. However, a potential explanation could be the structure of the exercise protocol, that encompassed the AEL only in the first repetition, allowing an increase in the eccentric power but not in the concentric power [6]. Consequently, the different loadings and exercises for AEL should be further investigated, since the available evidence on the effects of AEL is still not conclusive, and contrasting results are evident, due to different experimental protocols applied. In particular, the effectiveness of AEL as a form of conditioning activity to induce PAPE has not been fully elucidated. Moreover, there is a lack of evidence for the potentiation effects of a conditioning activity the days after its execution. Therefore, the purpose of this study was to investigate the effect of an AEL protocol, inducing the PAPE, on muscle power in volleyball players. It was hypothesized that AEL a) could elicit a greater PAPE and enhance subsequent jumping performance, force production, and rate of force development compared with TR loading; and b) the potentiating effects of AEL could last longer (up to 48 h) than TR loading.

## 2. Materials and Methods

### 2.1. Study Design 

A randomized clinical trial was used for the comparison between an AEL and a TR exercise protocol to evaluate whether the AEL, as a form of conditioning activity to induce PAPE, could induce greater and longer potentiating effects on subsequent performances of muscle power. To avoid misunderstandings but considering the controversy in the use of terminology and understanding of concept [24,25], in the current study it is always referred to as PAPE.

**Figure 1:**
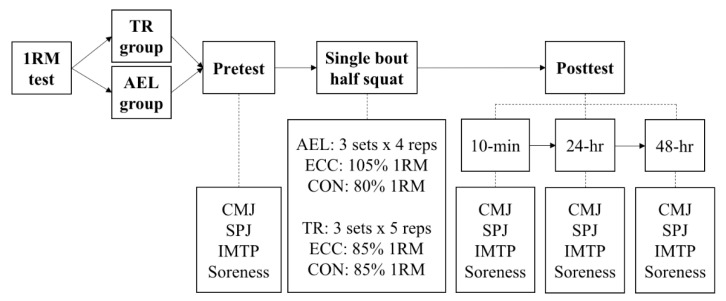
Study design. 1RM = one repetition maximum; AEL = accentuated eccentric loading; TR = traditional resistance training; reps = repetitions; ECC = eccentric phase; CON = concentric phase; CMJ = countermovement jump; SPJ = spike jump; IMTP = isometric mid-thigh pull.

After the execution of a 1-repetition maximum (1RM) test, participants were randomly assigned to the AEL or TR group for the execution of the exercise protocol, consisting of a half squat. Pre- (Pretest) and post- (Posttest) exercise protocol measurements included Countermovement Jump (CMJ), Spike Jump (SPJ), Isometric Mid-Thigh Pull (IMTP), and muscle soreness. Posttest measurements were repeated at 10 min (10-min), 24 h (24-h ), and 48 h (48-h ) (Figure 1).

This study was approved by the University of Taipei Institutional Review Board (Taipei, Taiwan, reference number: IRB-2020-054). All participants gave their informed written consent, and all the experimental procedures were conducted in accordance with the Declaration of Helsinki [26].

### 2.2. Participants

Sixteen male volley players were recruited from the men’s volleyball team from University of Taipei, according to the following inclusion criteria: (a) age 18–25 years; (b) experience in resistance training for at least two years. Exclusion criteria were: (a) presence of known cardiovascular, pulmonary, metabolic, bone, or joint diseases; (b) muscle and joint injuries during the last six months, (c) being a setter or libero, and (d) use of ergogenic aids and supplementations in the last six months. They were requested to maintain their normal nutritional habits for the entire duration of the study without the use of ergogenic aids and supplementation. Therefore, they were randomly assigned to the AEL group (*n* = 8, age = 21 ± 1.6 years, height 178.1 ± 8.2 m, body mass = 77.2 ± 13.1 kg, training experience = 10.4 ± 1.8 years, relative 1RM 146.3 ± 35.2 kg) and TR group (*n* = 8, age = 22 ± 1.6 years, height 180 ± 5.4 m, body mass = 77.2 ± 3.2 kg, training experience = 10.1 ± 1.5 years, relative 1RM 151.9 ± 31.5 kg).

### 2.3. Procedures

Participants reported to the laboratory on five occasions at the same time of the day (10:00 ± 30 min), with temperature and humidity kept consistent at 23 ± 1° C and 55 ± 5%, respectively. They were required to abstain from exercise during the 72 h prior the second and third experimental session. After ascertaining the inclusion criteria, participants were familiarized with all the experimental procedures during the first experimental session. The second experimental session was aimed at evaluating the lower limb maximal strength. During the third experimental session, the entire exercise protocol with the Pretest and Posttest measurements was executed, whilst the fourth and fifth experimental session was used to perform only the Posttest measurements at 24-h and 48-h. For all the experimental sessions, participants were required to avoid any form of exercise, to maintain their normal nutritional intake and hydration levels, and to abstain from alcohol and caffeine consumption during the 12 h prior to the sessions.

#### 2.3.1. Maximal Strength Testing

Lower limb maximal strength was determined by the 1RM back squat relative to body mass (BM) in kilograms (kg·BM^−1^). According to standard procedures [27], the protocol was performed using an Olympic barbell and a power half squat rack. After a standardized general warm up with 5-minute running activity on a treadmill followed by dynamic stretching exercises, a specific warm up included a set of 10 repetitions with 50% of an estimated 1RM (according to the subjects’ previous test or perceived capacity), a set of 5 repetitions with 75% of the estimated 1RM, and a final set of 1 repetition with 90–95% of the estimated 1RM. After a 3–5-minute rest period, participants completed 3 to 5 1RM attempts with progressively heavier weights (∼5%), interspersed with 3–5 min rest intervals, until a 1RM was determined. The 1RM was determined when participants were unable to perform the next repetition with an increased load. Participants were instructed to adopt a shoulder width stance in keeping with their normal squat stance, descend in a controlled manner, avoid bouncing at the bottom position, maintain as near a vertical torso as possible, and feet always flat on the ground. Moreover, they were advised to lower the barbell until their knees reach 90°, by having the hamstrings touching a resistance band placed in the power half squat rank.

#### 2.3.2. AEL and TR Exercise Protocol

The exercise protocol consisted of a volume-equated half squat exercise for both groups. The AEL group performed 3 sets of 4 repetitions, with an eccentric load of 105% 1RM and a concentric load of 80% 1RM). Two spotters helped participants to remove and add the load before and after the execution of the eccentric phase of each repetition. The TR group performed 3 sets of 5 repetitions (eccentric and concentric load with 85% 1RM). Participants were asked to lift the barbell upward to complete the concentric phase. Repetitions were interspersed by 3 seconds of recovery, required to reload the barbell for the following eccentric phase of each repetition for the AEL group, whilst a 3-min rest interval between sets was allowed. The experimental session was executed 72 h after the previous session for the determination of 1RM. Participants executed a standardized general warm up with 5-minute running activity on a treadmill followed by dynamic stretching exercises and a specific warm up including a set of 5 repetitions with 20% of 1RM followed by 3 repetitions at 50% of 1RM.

#### 2.3.3. Countermovement Jump Test

Participants performed the CMJ test on a dual force-plate (OR6-7-OP, Advanced Mechanical Technology, Inc., Watertown, MA, USA) with a sampling frequency of 1000 Hz, at Pre, 10-min, 24-h, and 48-h after the exercise protocol. Each participant performed two trials, with a three-minute rest interval in between. Each CMJ trial began with the participant standing upright, then descending to a depth of approximately 90° knee angle, and then immediately jumping upward with maximum effort [28]. All participants were required to bear a light barbell (350g) on the posterior shoulder to eliminate the influences of arm swing on during each jump [7]. Jump height was calculated from the “flight” time between take-off and landing [29]. Peak velocity was calculated from the initiation of concentric phase to the phase before take-off. The rate of force development (RFD) was calculated as between the concentric peak force (PF) and the baseline force value during the concentric phase. The mean of all dependent variables from two CMJ trials was retained and used for statistical analysis.

#### 2.3.4. Spike jump test

Participants performed the SPJ test using the vertec device (Vertec Jump Trainer, Jump USA, USA) at Pre, 10-min, 24-h, and 48-h after the exercise protocol [30]. Each participant performed three trials, with a three-minute rest interval in between. During the approach phase, participants approached with “left foot, right foot-left foot, jump” if participant was right-handed, and vice versa if he was left-handed. They flexed their legs with self-selected depth and performed an overhead arm swing to jump as high as possible with maximum effort to hit the vertec vanes by using their dominant hand. The mean of jump height of three SPJ trials was used for statistical analysis.

#### 2.3.5. Isometric Mid-thigh Pull Test

Participants performed the IMTP on a dual force-plate (P6000, BTS Bioengineering, Milano, Italy) with a sampling frequency of 1000 Hz, at Pre, 10-min, 24-h, and 48-h after the exercise protocol. Participants performed three maximal IMTP trials, with a three-minute rest interval in between. They were secured to the immovable bar by using lifting straps and athletic tape to prevent their hands from slipping, and the bar placed equidistant at the mid-point between the iliac crest and patella [31]. The knee angles were set within 125 ± 5° (full extension = 180°), while the hip angle was set at approximately 145° [32]. Hip and knee angles were confirmed using a goniometer. Once positioned, the trial began after a countdown “3, 2, 1, Pull”, and participants were required to pull the bar vertically with maximum effort for five seconds, and strong verbal encouragement was provided [31]. The ground reaction force data were collected to obtain the relative PF and RFD. The maximum force generated during each IMTP trial was reported as PF. The RFD was calculated as a difference between the PF and the baseline force from the initiation of the pull during each IMTP trial. The mean of all dependent variables of three IMTP trials was retained and used for statistical analysis.

#### 2.3.6. Muscle Soreness Test

The muscle soreness test was performed at Pre, 10-min, 24-h, and 48-h after the exercise protocol. Participants were required to rate the quadriceps and hamstring soreness perception after one trial of the test. The Visual Analog Scale (VAS) consisted of a 100-mm continuous line with “no pain at all” on one side (0 mm) and “extremely painful” on the other side (100 mm) [33]. Each participant began by stepping on the 30-cm aerobic step with one leg, then standing upright on the aerobic step for 1 second, then stepping off the aerobic step with one leg and landing on the floor. Participants then noted the perceived muscle soreness on the VAS. The muscle soreness test was performed at the beginning of the experimental session before any other test to avoid the influences of fatigue caused by the other tests.

### 2.4. Statistical Analyses

Mean ± SD was used to describe all dependent variables. All statistical tests were conducted using the Statistical Package for the Social Sciences, version 25.0 (SPSS Inc., Chicago, IL, USA) with statistical significance set at *p* ≤ 0.05. The normality assumption for each variable was verified using the Shapiro–Wilk test, which confirmed the normal distribution of data. To avoid individual variation, CMJ height, peak velocity, and RFD, SPJ height, and IMTP peak force and RFD were normalized to the percentage of the premeasurement (posttest/pretest) × 100% for comparison. The results were shown in percentage (%) ± standard deviation (%). A prior evaluation of homogeneity of variance and sphericity was conducted using Levene’s test and Mauchly’s test, respectively. Consequently, a mixed analysis of variance (ANOVA) was applied to examine the changes of each variable over time between the two groups, considering the within-subjects factor time (Pre, 10-min, 24-h, and 48-h ) and the between-subjects factor group (AEL and TR). Effects sizes for main effects were calculated as partial eta squared (𝜂𝑝^2^) and interpreted as small (0.01–0.06), medium (0.06 < 𝜂𝑝^2^ < 0.14), and large effects (>0.14) [34]. In case of significant interactions, pairwise comparisons (t-test) with Bonferroni adjustments were applied. Cohen’s d effect sizes (ESs) [35,36] were calculated for each pairwise comparison and interpreted as trivial (<0.19), small (0.20–0.59), moderate (0.60–1.19), large (1.20–1.99), very large (2.0–4.0), and extremely large effects (>4.0).

## 3. Results

Descriptive statistics (Mean ± SD) for the investigated variables are presented in Table 1. 

**Figure 2:**
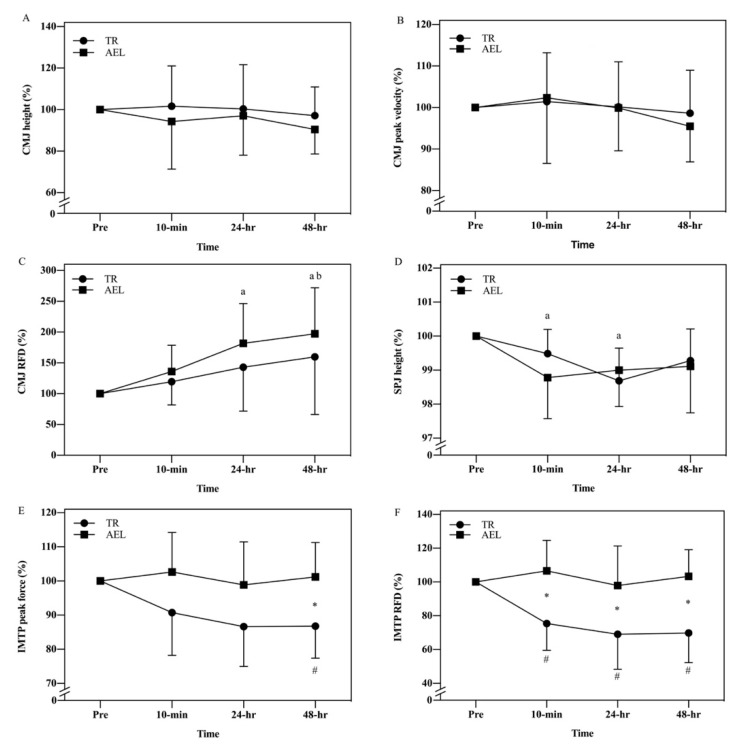
Normalized changes (expressed in percentage) in countermovement jump (CMJ) height (**A**), CMJ peak velocity (**B**), CMJ rate of force development (RFD) (**C**), spike jump (SPJ) height (**D**), isometric mid-thigh pull (IMTP) peak force (**E**), IMTP RFD (**F**) between accentuated eccentric loading exercise (AEL) and traditional resistance loading exercise (TR) group. * Denotes significant differences between AEL and TR. # Denotes significant differences from Pre in TR group. a Denotes significant main effect of time and post hoc differences from Pre. b Denotes significant main effect of time and post hoc difference from 24-h.

For CMJ height and peak velocity, no main effects of time, group, and interaction emerged (Figure 2A-B). Regarding CMJ RFD (Figure 2C), only a main effect of time was found (*p* = 0.003, 𝜂𝑝^2^ = 0.335). Post hoc analysis maintained differences for Pre compared with 24-h (*p* = 0.025, Cohen’s d = 1.213) and 48-h (*p* = 0.014, Cohen’s d = 1.322), and between 24-h and 48-h (*p* = 0.035, Cohen’s d = −0.241). Similarly, only a main effect of time emerged for SPJ height (*p* < 0.001, 𝜂𝑝^2^ = 0.351) (Figure 2D). Post hoc analysis maintained differences for Pre compared with 10-min (*p* = 0.021, Cohen’s d = 1.2) and 24-h (*p* = 0.003, Cohen’s d = 1.65).

A time by group interaction (*p* = 0.042, 𝜂𝑝^2^ = 0.175) emerged for IMTP peak force. Pairwise comparisons with Bonferroni correction (*p* < 0.0083) showed that peak force significantly decreased from Pre to 48-h post (*p* = 0.005, Cohen’s d = 1.993) in TR group only. Peak force for TR group was significantly lower than that for AEL group at 48-h (*p* = 0.01, Cohen’s d = 1.479) (Figure 2E). 

A time by group interaction (*p* = 0.005, 𝜂𝑝^2^ = 0.265) was found for IMTP RFD. Pairwise comparisons with Bonferroni correction (*p* < 0.0083) showed that RFD significantly decreased from Pre to 10-min (*p* = 0.003, Cohen’s d = 2.179), from Pre to 24-h post (*p* = 0.004, Cohen’s d = 2.111), and from Pre to 48-h post (*p* = 0.002, Cohen’s d = 2.421) in TR group. RFD for TR group was significantly lower than that for AEL group at 10-min (*p* = 0.003, Cohen’s d = 1.83), 24-h (*p* = 0.021, Cohen’s d = 1.308), and 48-h (*p* = 0.001, Cohen’s d = 2.002) (Figure 2F).

**Figure 3:**
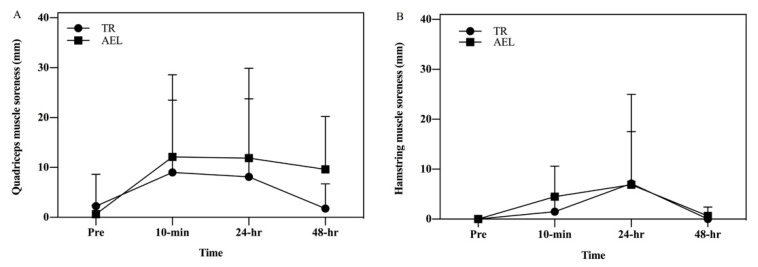
Comparisons of quadriceps (**A**) and hamstring (**B**) muscle soreness between accentuated eccentric loading exercise (AEL) and traditional resistance loading exercise (TR) group.

For changes in quadriceps (*p* = 0.1809) and hamstring (*p* = 0.9016) muscle soreness, no main effects of time, group, and interaction emerged (Figure 3A,B).

## 4. Discussion

This study intended to advance scientific knowledge on the effectiveness of an AEL protocol to induce PAPE in volleyball players and on the prolonged potentiating effects the days after the exposure to the conditioning activity. In particular, the ability to produce force in a short period of time is the critical factor for the success in sport performances [1]. Therefore, the AEL exercise protocol proposed by the current study can be considered an effective strategy to maintain high force production during the IMTP. Conversely, a traditional resistance training protocol caused an impairment in the performance of the IMTP. The difference in the results could be attributed to the additional load prescribed during the eccentric phase (105% 1 RM), which elicited potentiating effects on the subsequent performance. 

The findings of the current study are similar to the those proposed by Ojasto and Häkkinen, where the eccentric overload prescription elicited an unfavorable effect on maximal strength expression, likely because of fatigue [9]. A brief period of repetitive stimulation results in enhanced contractile response (potentiation) while continued stimulation results in impaired or attenuated contractile response (fatigue) [37]. PAP has been attributed mainly to two sources. The first is the phosphorylation of the regulatory light chains during the previous contraction. This phosphorylation alters the structure of the myosin fibers in the muscle, modifying the state of the crossed bridge of actin-myosin and producing more sensitivity for the release of Ca^2^+ to the sarcoplasmic reticulum [38]. The other explanation is neurological: it has been observed an increased motor neurons excitability during the contraction produced by PAP. However, since the effect of PAP would dissipate over 4–6 min after induced [18], the results of the current study cannot be explained by this neurological factor. 

In the current study the greater intensity of AEL exercise protocol during the eccentric phase might elicit better potentiating effects on concentric performance. During the eccentric action the enhancement of elastic energy storage in the muscle fibers and tendons can contribute to a greater generation of force during the concentric contraction [39]. Because of the elastic nature of tendons, the additional force present at the start of the concentric phase, following the stretch during the eccentric phase, results in relatively greater tendinous extension with less myofibrillar displacement. Similarly, in a CMJ test with greater joint moments observed at the start of the upward movement caused by the effect of stretch-shortening cycle. Therefore, both IMTP RFD and PV of the AEL group could remain at the same level of the baseline measurement. The reason for RFD enhancement may be the increased motor unit recruitment during the eccentric phase [40]. Less activation was needed for recruitment once a motor unit was recruited [41], hence the AEL load proposed by the current study may have recruited larger motor units during the eccentric phase and they remained activated during the initiation of the concentric phase, thus enhancing the RFD performance. 

It is generally accepted that the effect of PAPE could last for 12 min [42]. However, the current data demonstrated long-lasting effects on IMTP peak force, IMTP RFD, and CMJ RFD until 48 h. Sport participation exposes athletes to demanding trainings or competitions leading to cumulative fatigue lasting over a 5-day period [43]. Conversely, the potentiating effects of AEL seem to be elicited to counterbalance the fatigue at least for 48 h, which could be translated into implications for the training prescription (possibility to increase the training volume) and return to play (anticipate) strategies. Although the IMTP test is based on isometric contraction, the potentiating effects of AEL seems to benefit the isometric contraction in counterbalancing the fatigue. It is important to highlight that the enhancement of IMTP was significantly correlated with CMJ, squat jump [44], sprint, agility [45], weightlifting movements, squat, and deadlift [46], hence can be considered an important determinant of sport performance. Moreover, SPJ performance demonstrated a decrement until 24 h after the execution of both AEL and TR exercise protocols, without any difference between protocols. However, Sheppard et al. found that SPJ height could be improved by assisted jumping training with 10 kg loading for 5 weeks [30]. The possible reasons for this discrepancy could be the muscle fatigue induced by the high resistance training load. Although Rassier and Maclntosh reported that potentiation and fatigue could coexist even if they have opposing effects on force production in skeletal muscle [37], further research is still necessary to elucidate the exact balance between negative and positive effects of a prior exercise on subsequent force production relative to sport performance.

The current study did not demonstrate a differential effect of the two exercise protocols on CMJ and SPJ parameters. Regarding CMJ height and peak velocity, the current results are in contrast with the previous findings demonstrating an effect of AEL protocols in improving jumping height [20,47,48,49]. The possible explanations for the contrasting results could be attributed to the intensity of loads, repetitions, and the PAP time window. Aboodarda et al. implemented the eccentric overload of 20 and 30% of body mass during the eccentric phase with 3 CMJ trials for each condition [49]. Beyond that, Sheppard et al. used absolute loads of 20 kg during the eccentric phase with accentuated eccentric load block jump for AEL condition [22]. The large differences in loads and repetitions may have induced greater potentiating effects due to less fatigue compared to the 105% of 1RM during the eccentric phase with three sets of four repetitions of the current study. Bridgeman et al. found that a protocol of 20% of body mass with one set of five drop jumps resulted in improving the CMJ height compared with free body mass and 10% of body mass [20]. Moreover, CMJ height at 2 and 6 min post measurements was greater than 12 min post. These results might be in contrast with the current study because the PAP time window would disappear after 10 min. Therefore, PAPE would be more suitable to explain the findings of the current study [42].

Considering CMJ RFD, it has been previously demonstrated a lack of effect after a squat exercise protocol with three different eccentric overloads (105, 110, and 120% of concentric 1RM) [21]. Furthermore, Wagle et al. also found that the concentric RFD was not potentiated by using a load of 105% 1RM during the eccentric phase [6]. Merrigan et al., using a back squat protocol (3 sets × 5 repetitions at eccentric/concentric), found that a single supramaximal eccentric phase of 120% 1RM increased subsequent velocity and power with concentric loads of 65% 1RM, but not 80% 1RM. A Large relative difference between the eccentric and concentric load could have induced beneficial effects during the concentric phase of back squat [50]. In light of the available evidence, it is not possible to derive definitive conclusions and further research is necessary to elucidate the potentiating effects on CMJ performance.

Muscle soreness of quadriceps and hamstring was not significantly elevated compared with baseline for both AEL and TR exercise protocol. This result is contrary to the findings from Hackney et al. where a peak muscle soreness at 24 h after exercise was found [37]. However, Curty et al. observed no change in muscle soreness at 24-h, 48-h, and 72-h after 130% of 1RM free weights unilateral elbow extension with 10 repetitions [51]. Therefore, the proposed AEL can be considered suitable for athletes engaged in consecutive training sessions since it does not cause severe soreness, similar to the TR protocol. 

Despite promising results, this study has some limitations that need to be addressed and can serve as guidance for future research. The limited number of participants and only male gender is relative to the availability of the sole collegiate men’s volleyball team. The inclusion of athletes from different teams would have increased the sample heterogeneity in terms of training and competitions scheduled. This study aimed to investigate the long-lasting potentiating effects 10 min after the conditioning activity, hence the change of the PAP effect that occurred within 10 min could not be investigated. A single AEL protocol has been proposed, having potentiating effects only on IMTP peak force and RFD. Therefore, further research should compare AEL protocol with different eccentric loadings and different combinations of the training protocols to balance the coexistence of potentiating and fatigue effects. The cluster sets designed by Wagle et al. were considered to allow athletes more resting time to overcome the fatigue [6]. Besides, future research should be aimed to implement 120% 1RM for eccentric phase, which was applied from several studies [17,20,21].

## 5. Conclusions

The main finding from this study is a retention of the ability to produce a high amount of force in a short period of time by accentuated eccentric loading. The effects of PAPE could last not only 10 min but also 48 h, which could be translated into implications for the training prescription (possibility to increase the training volume) and return to play (anticipate) strategies. The different dose and relative differences between the eccentric and concentric load should be further analyzed to overcome the accumulation of fatigue. 

## Figures and Tables

**Table 1:** Mean ± SD for CMJ height, peak velocity, RFD, SPJ height, IMTP peak force and RFD, and muscle soreness before (Pre), 10 min (10-min), 24 h (24-h ), and 48 h (48-h ) after a single bout of half-squat exercise for AEL and TR group.

	Pre	10-min	24-h	48-h
CMJ Height (cm)				
AEL	35.06 ± 4.14	33.01 ± 8.80	34.08 ± 7.90	31.70 ± 5.46
TR	35.91 ± 7.15	35.79 ± 5.66	35.33 ± 6.26	34.19 ± 4.11
CMJ PV (m/s)				
AEL	2.81 ± 0.23	2.87 ± 0.48	2.80 ± 0.33	2.68 ± 0.30
TR	2.87 ± 0.27	2.90 ± 0.26	2.86 ± 0.24	2.81 ± 0.18
CMJ RFD (N/s)				
AEL	2946.38 ± 761.71	3370.96 ± 883.47	4319.09 ± 3122.46	4517.08 ± 2650.05
TR	2642.96 ± 767.05	3449.13 ± 944.01	4521.40 ± 1670.13	4889.81 ± 1714.18
SPJ Height (cm)				
AEL	317.75 ± 15.54	315.09 ± 13.81	313.06 ± 14.75	314.71 ± 13.58
TR	314.61 ± 15.46	312.07 ± 14.91	312.88 ± 15.17	311.27 ± 13.26
IMTP PF (N)				
AEL	3143.47 ± 528.24	2915.02 ± 366.81	2893.59 ± 666.10	2764.42 ± 449.96
TR	2993.5 ± 386.24	3026.97 ± 490.84	2834.33 ± 459.97	2866.66 ± 357.58
IMTP RFD (N/s)				
AEL	331.41 ± 17.65	307.51 ± 15.56	308.23 ± 16.76	308.50 ± 14.87
TR	315.33 ± 10.01	313.69 ± 9.82	311.15 ± 9.22	313.00 ± 8.51
Quadriceps soreness (mm)				
AEL	0.62 ± 1.77	12.13 ± 16.47	11.88 ± 18.01	9.63 ± 10.60
TR	2.25 ± 6.36	9.00 ± 14.50	8.13 ± 15.64	1.75 ± 4.95
Hamstring soreness (mm)				
AEL	0.00 ± 0.00	4.50 ± 6.09	6.88 ± 10.64	0.00 ± 0.00
TR	0.00 ± 0.00	1.50 ± 3.21	7.13 ± 17.85	0.00 ± 0.00

Note: AEL = accentuated eccentric loading; CMJ = countermovement jump; IMTP = Isometric mid-thigh pull; PF = peak force. PV = peak velocity; RFD = rate of force development; SPJ = Spike jump; TR = traditional training.

## Data Availability

The data presented in this study are available on request from the corresponding author. The data are not publicly available due to privacy.
